# Editorialets

**Published:** 1911-03

**Authors:** 


### Editorialets.

### OUR OWN HORN.

A Voice from "Old Virginy."—It is a pleasure, my dear friend, to know that *you know* what a penalty all good men must pay for being honest and fearless in their fight for principle. God knows that you and I have never flinched in our work nor traded principle for pelf. God bless you, my dear, honest, brave friend. I was aware of the fact that you are a Virginian, and you have long ago proven that you represent the best element of the Old Dominion's illustrious sons.

Bryce (*Southern Clinic*),
Richmond, Va.

I value the "Red Back," no little, for it is the most welcome visitor to my studio of all the valuable journals I take; always filled with fresh, valuable matter;—fine selections and original contributions, rounded out by editorial embellishments that are unsurpassed in excellency by those of the best journals of the time. Be good enough, dear doctor, to continue to send it to me, for it is highly interesting and instructive—a little gem in its way—therefore, I rank it among "The friends I have and their adoption tried."

With sincere wishes for your success and happiness, and the same for your estimable wife, I remain, faithfully,

Your friend,
S. Eagon,
Dallas, Texas.

*Dear Dr. Daniel:*

One of the pleasantest episodes of my advertising life is the privilege of writing you once a year renewing our contract with the old "Red Back." I hope to have that pleasure for many years to come, for naturally we both want to live at least twenty-five or thirty years more, and if you keep on publishing as good a JOURNAL as you have for the last two or three hundred years, and I am still able to hold on to my present job, you will probably hear from me about this time every year for the next century or two.

Joking to one side, my dear doctor, it gives me pleasure to renew our contract for another year.

With best wishes for your continued success, I am,

Very truly yours,

H. M. SEEM,

Manager Sharp & Dohme, New York and Baltimore.

[Twenty-seventh year.—ED.]

I can't keep office without the "Red Back."

J. C. HOLMAN,

Franklin, Texas.

A REAL RIP VAN WINKLE.

THE STRANGE CASE OF DR. BRUNO. By F. E. Daniel, M. D. Fourteen illustrations. Von Boeckmann-Jones Company, Austin, Texas. Price, $1.50, or with "Red Back" one year $2.00.

The editor of the TEXAS MEDICAL JOURNAL, Dr. Daniel, has written one of the most fascinating books of the century. The book is remarkable, not only as a love story in which the talented author describes most vividly the vibrations of the *chrordae tendinae,* but he also delves deeply into the science of the phenomena of life—biology, cytology, physiological chemistry, electro-dynamics, psychology, amnesia (double personality) and suspended animation. Never was there written a more beautiful, weird, scientific, tragic love story. It pulses with manly, chaste, soul-stirring events of intense human and scientific interest.

From Literary Department, *Pacific Medical Journal,* by Winslow Anderson, A. M., M. D., M. R. C. P., London; M. R. C. S., England; L. S. A., London, etc.; Professor of Gynecology and Abdominal Surgery in the College of Physicians and Surgeons of San Francisco, etc., Editor-in-Chief.

PAYS FIVE YEARS IN ADVANCE.—Dr. R. G. Lloyd, of Royse, Texas, sends the "Red Back" a five—renewing his subscription to September, 1915, as "a token of appreciation," he says.

Such tokens are most gratefully received. They serve to stimulate to renewed effort, to please and instruct, and strengthen an arm tired striking at the evils and wrongs that beset the medical profession.

Instructive and delightfully entertaining.

J. A. MATTHEWS,
Garner, Texas.

I have been reading the "Red Back" since 1885, and could not think of giving it up at this late day. It is a journal fearless in the advocation of what is right, and of cleanness in the profession. It asks no concessions; it makes none.

With best wishes for the JOURNAL and its editor, I am, very sincerely,

Your friend,
J. T. BENBROOK,
Rockwall, Texas.

PAYS IN ADVANCE TO 1918.—Dr. Isaac E. Clark, of Schulenburg, Texas, always pays five years in advance for the "Red Back." He has done so for more than a quarter of a century. He was already paid to January, 1913, when he handed me a five one day last week during a visit to Austin. There are few men like him, "True and tried," in the language of Polonius, I "grip him to my heart with hooks of steel," or words to that effect.

*Dear Doctor:*

Enclosed find check to advance my subscription to the "Red Back." I also want to express my appreciation of your efforts in giving us the JOURNAL, after being a reader of it for nearly twenty-five years. No doubt you have made some mistakes, as all do, but you have given us an able, clean, entertaining publication. So, as a believer that bouquets should be for the living, I offer you this tribute to your efforts in the cause to elevate mankind.

Sincerely yours,
J. Q. BURTON,
Farwell, Texas.

---

MISCELLANEOUS.—The Texas State Board of Medical Examiners will hold its regular semi-annual meeting at Austin, in the hall of the House of Representatives, June 27, 28, 29, 1911. J. D. Mitchell, Secretary, Fort Worth, Texas. If interested, write to him for details.

---

IN INDIANA.—A bill providing for a certificate of freedom from venereal infection, before a marriage license may be issued (the Bedgood bill), passed the Indiana House February 9th (ult.).

